# 

**Published:** 1899-05-06

**Authors:** 


					CADBURY'S Ft* M CADBURY'S
COOOA % M M JgL COCOA
ABSOLUTELY PURE. -1 ^0^7 NO DRUGS OR ALKALIES USED.
W\
'Rtf/CH HBSPITAU
No. 658. vJVol. XXVI. [SspfpeS Saturday,
May Gin, 1899.
[^bscription Terms,] pRICE TWOPENCE.

				

